# Risk factors of shunt-dependent hydrocephalus after subarachnoid hemorrhage: a systematic review and meta-analysis based on observational cohort studies

**DOI:** 10.1007/s10143-024-02589-5

**Published:** 2024-08-13

**Authors:** Lingzhuo Chen, Yichen Meng, Qiang Xue, Yuanyu Zhao, Xuhui Zhou, Kejia Hu, Hua He

**Affiliations:** 1 Departments of Neurosurgery, The Third Affiliated Hospital, Naval Medical University, Shanghai, P.R. China; 2 Departments of Orthopaedics, The Second Affiliated Hospital, Naval Medical University, Shanghai, P.R. China; 3 Department of Neurosurgery, Ruijin Hospital, Shanghai Jiao Tong University School of Medicine, No. 197 Second Ruijin Street, Shanghai, P.R. China; 4 Center for Functional Neurosurgery, Ruijin Hospital, Shanghai Jiao Tong University School of Medicine, Shanghai, P.R. China; 5 Organ transplantation, The Second Affiliated Hospital, Naval Medical University, Shanghai, P.R. China

**Keywords:** Risk factor, Shunt-dependent hydrocephalus, Subarachnoid hemorrhage, Meta-analysis

## Abstract

Shunt dependent hydrocephalus (SDHC) is a common sequel after aneurysmal subarachnoid hemorrhage (aSAH) and factors contributing to the development of SDHC remain obscure. The aim of this study was to identify predictors of SDHC following aSAH. We conducted a systematic review based on the Meta-analysis of Observational Studies in Epidemiology (MOOSE) guidelines. We searched electronic databases including Pubmed, Embase, and Cochrane databases from 1980 through August 2019 for studies on the risk factors of SDHC after aSAH. Inclusion criteria were: (1) SAH and hydrocephalus confirmed by CT or magnetic resonance imaging findings; (2) the odds ratios (ORs) or the relative risk (RR) with 95% confidence interval (95%CI; or crude data that allowed their calculation) were reported; and (3) languages were restricted to English and Chinese. Two independent authors collected the data including study design, characteristics of patients and potential risk factors. Random-effects models were used to estimate weighted mean differences (WMD), relative risks (RR) with corresponding 95% confidence intervals (CI). For analysis with significant heterogeneity, subgroup analyses stratified by study design and geographic area were performed. In all, 37 cohort studies met inclusion criteria. Several factors were associated with SDHC. Infection, acute hydrocephalus, placement of external ventricular drainage, older age, higher Hunt and Hess grade, intraventricular hemorrhage, rebleeding, and mechanical ventilation were associated with greater 2-fold increased risk of SDHC. Vasospasm, female gender, high Fisher grade, preexisting hypertension, aneurysm in posterior location and intracerebral hemorrhage were associated with less than 2-fold increased risk. Treatment modality and diabetes mellitus were not associated with SDHC. SDHC is a multi-factorial disease that is associated with patient and treatment factors. Acknowledgement of these potential factors could help prevent SDHC.

## Introduction

Hydrocephalus is a frequent complication after aneurysmal subarachnoid hemorrhage (aSAH), incidence ranging from 6 to 67%. The pathophysiological mechanism of hydrocephalus after aSAH is still obscure, which results in difficulties with intervention based on etiological factors. Although V-P shunt or other CSF diversion operations is therapeutic in some patients, additional surgical procedures are required, which are accompanied by risk of infection, duct obstruction, and implant rejection. Thus, it is important to find the risk factors that predict shunt dependent hydrocephalus (SDHC). Different factors have been proposed to predict SDHC, including age, gender, pre-existing medical comorbidities, admission status, in-hospital status, treatment modality, post-treatment complications such as intracranial infection, location of aneurysms, external diversion [1], treatment modality [2] and markers of inflammation such as IL-6 [3] and TGF-beta [4]. Some other factors, such as size of the aneurysm are controversial [5]. We conducted this systematic review and meta-analysis to identify factors that predict SDHC after aSAH.

## Methods

### Search strategy

This systematic review and meta-analysis was based on the Meta-analysis of Observational Studies in Epidemiology (MOOSE) guidelines [6]. A computerized search of Pubmed, Embase, and the Cochrane databases (from 1980 through August 2019) was performed for studies on the risk factors of SDHC after aSAH. We chose the year 1980 as the start to maximize the reliability of included articles because most medical centers did not have the means to diagnose aSAH using computed tomographic (CT) scans until the late 1970s. The following terms were used in different combinations: hydrocephalus, chronic hydrocephalus, shunt-dependent hydrocephalus, SDHC, aneurysmal subarachnoid haemorrhage, cerebral aneurysm, intracranial aneurysm, and risk factor. Manual search was also conducted to identify relevant publications from the citation lists of the included articles.

### Inclusion and exclusion criteria

Studies were included if they met the following criteria: (1) the study was a randomized controlled trial or a prospective or retrospective cohort and case-control study; (2) SAH and hydrocephalus confirmed by CT or magnetic resonance imaging findings; (3) the odds ratio (OR) or the relative risk (RR) with 95% confidence interval (95%CI; or crude data which allowed their calculation) were reported; and (4) the language was restricted to English and Chinese.

The following types of studies were excluded: (1) case series, case reports, reviews and descriptive studies; (2) reports with a small number of participants (less than 20 patients); (3) SAH was confirmed without aneurysms being found; (4) the crude data was not given or the OR or RR could not be calculated; and (5) publications were not written in English or Chinese.

### Data extraction and study quality assessment

Two authors independently read the full texts and extracted relevant data from each publication. The following data were extracted: first author, year of publication, country, research type, shunt related factors, number of participants and male/female ratio. Shunt related factors including age, gender, Hunt-Hess grade, Fisher grade, treatment modality, intraventricular hemorrhage, intracerebral hematoma, intracranial infection and symptomatic vasospasms were also extracted. The data were independently extracted by two authors, and disagreements were resolved by consensus.

The quality of the included studies was assessed by two independent reviewers using the Newcastle-Ottawa scale (NOS) for case-control and cohort studies [7]. This scale evaluates studies from three perspectives: selection, comparability and outcome (cohort studies) or exposure (case-control studies). A study was awarded a maximum of four points for selection, two points for comparability and three points for outcome or exposure. The total NOS score was nine points. In our meta-analysis, a study with more than six points was considered a high quality study; otherwise it was considered a low quality study.

### Statistical analysis

Statistical analyses were performed using Review Manager Version 5.3 (Cochrane Collaboration, Software Update, Oxford). We analyzed the RRs with 95%CIs for dichotomous variables and the weighted mean difference (WMD) with 95%CIs for continuous variables. Adjusted risk estimates were also abstracted. The unadjusted risk estimates were calculated using original data when the adjusted risk estimates were unavailable. The *I*-squared (*I*^*2*^) statistic was used to assess statistical heterogeneity among studies. *I*^*2*^ > 50% reflected significant heterogeneity [8]. Random-effects models were used to pool the data. The robustness of the combined results was evaluated by sensitivity analysis were the analysis was repeated after excluded one study at a time [9]. Funnel plots were used to assess publication bias in analyses that included more than 10 studies.

## Results

A total of 2220 articles were identified after the initial search. After exclusion of 1,795 articles that were irrelevant to our research, 425 remained. In total, 90 articles were retrieved for full-text review and 40 studies were identified. Finally, three of them were excluded because the crude data for the risk factors of interest could not be extracted. Therefore, 37 studies matched the inclusion criteria and were chosen for the following meta-analysis (**Supplement 1**) [2, 10–45].

These studies dated from 1992 to 2019 with retrospective or prospective nonrandomized designs and included a total of 36,295 subjects, of which 3828 (10.55%) developed SDHC (Table 1). Among these, 22 studies using multivariate analysis to explore independent risk factors provided adjusted risk estimates [2, 11, 13, 15–17, 19, 21, 23, 27, 32, 35–45]. Of the 37 included studies, eleven originated from Europe (German, Finland, Switzerland, Norway, Austria and Sweden) [2, 11, 13, 14, 22, 24, 26, 29, 34, 36, 37], seventeen from Asia (China, Korea and Japan) [10, 15, 16, 18–20, 25, 28, 30–32, 35, 38–42], one from Australia [17] and eight from America (Canada and United States) [12, 21, 23, 27, 33, 43–45]. Quality assessment showed all included studies were of high quality with a mean score of 7.38 points (**Supplement 2**).

**Table 1:** Characteristics of the studies included in the analysis

Author, Year	Country	Design	Sample size	Mean age(range)	M/F	No. shunted	Potential factors reported	Adjusted
Saveland,1992	Sweden	Prospective cohort	325	53.5(9–84)	120/205	27	D, E,M	No
Tapaninaho,1993	Finland	Retrospective cohort	835	-	438/397	81	A, B,C, D,E, F,G, I,J	No
Pietila,1995	Germany	Retrospective cohort	204	-	61/143	30	A, C,D	No
Gruber,1999	Austria	Prospective cohort	187	-	65/122	40	A, B,C, D,E, F,J, K	No
Lin,1999	PRC	Retrospective cohort	141	47.4(20–88)	55/86	20	A, B,C, D,E, F,G, L,M, N	No
Yoshioka,2000	Japan	Retrospective cohort	576	-	-	215	A, C,D, E,F, G,H, L	No
Dorai,2003	US	Retrospective cohort	718	53.2(17–89)	239/479	152	A, B,C, D,E, F,G, K,L	No
Hirashima,2003	Japan	Retrospective cohort	114	58.0(24–86)	38/76	39	A, B,C, D,E, G,M, N,L, O,P	Yes
Dehdashti,2004	Switzerland	Prospective cohort	245	50.5(18–78)	88/157	38	A, B,E, G,H, K	Yes
Varelas,2006	US	Retrospective cohort	183	-	72/111	12	A, B,C, D,E, F,G, K,O	Yes
de Oliveira,2007	Germany	Prospective cohort	385	-	144/241	71	A, B,C, D,E, F,G, K	Yes
Kwon,2008	Korea	Retrospective cohort	734	53.0(17–82)	236/498	66	A, B,C, D,E, F,G, H,I, L	Yes
O’Kelly,2009	Canada	Retrospective cohort	3120	-	1112/2008	585	A, B,C, G,H, K,O, Q	Yes
Nam,2010	Korea	Retrospective cohort	736	55.1(21–86)	221/370	145	A, B,C, D,E, F,G, K	No
Rincon,2010	US	Prospective cohort	580	-	189/391	61	A, B,D, E,H, I,L, M,K, N,O, P	Yes
Wang,2012	PRC	Retrospective cohort	86	-	23/63	15	A, B,F, H,L, M,N, P	No
Lai,2013	Australia	Retrospective cohort	10,807	-	-	701	C, F,H, I,K, O,Q	Yes
Liu,2013	PRC	Retrospective cohort	129	-	64/65	16	A, B,C, D,E, I,M,	Yes
Woernle,2013	Switzerland	Prospective cohort	389	-	132/257	91	B, K	No
Yang,2013	PRC	Retrospective cohort	88	54.5(26–84)	25/63	22	A, B,C, D,E, F,K, L,O	No
Bae,2014	Korea	Retrospective cohort	215	56.5(27–79)	75/140	30	A, B,C, D,E, F,G, K,O	No
Erixon,2014	Norway	Prospective cohort	90	56.0(30–84)	26/64	30	A, B,C, D,E, K	Yes
Shao,2014	PRC	Retrospective cohort	136	51.4(25–79)	56/80	23	A, B,C, D,E, F,K	No
Yu,2014	PRC	Retrospective cohort	202	54.4(5–83)	81/121	40	A, B,C, D,E, F,G, I,J, K,L, M,N, O	Yes
Walcott,2014	US	Retrospective cohort	8889	-	3613/5276	116	A, B,I, O,Q	No
Zaidi,2015	US	Prospective cohort	471	53.72	139/332	147	A, B,C, F,H, K,M, P	Yes
Adams,2016	Finland	Retrospective cohort	1553	-	663/890	275	A, B,C, D,E, F,G, H,K, O	Yes
Tso, 2016	Canada	Prospective cohort	413	-	121/292	71	B, C,I, L,M, O	Yes
Na, 2017	Korea	Retrospective cohort	181	54.4(28–82)	75/106	43	A, B,C, D,E, F,H, O	Yes
Han, 2018	Korea	Retrospective cohort	447	54.7	159/288	80	A, B,C, D,E, F,H, L,	Yes
Koyanagi, 2018	Japan	Retrospective cohort	566	65(53–75)	144/422	127	B, C,F, G,H, L	Yes
Jeong,2018	Korea	Retrospective cohort	275	70.6	55/220	53	A, C,D, E,F, G,H, I,K, M,O, P	Yes
Paisan, 2018	US	Retrospective cohort	888	-	267/621	116	A, B,C, F,G, K,L, O	Yes
Diesing, 2018	Germany	Retrospective cohort	225	-	73/152	61	B, C,D, E,F, K	Yes
Lenski, 2019	Germany	Retrospective cohort	63	55.2(18–80)	21/42	21	A, B,C, F,K, L	No
Hao,2019	PRC	Retrospective cohort	845	-	317/528	121	A, B,C, D,F, K,M, P	Yes
Kim,2019	Korea	Retrospective cohort	254	55.3(22–90)	74/180	47	A, B,C, F,K, L,M, P	Yes

A: age; B: gender; C: ruptured aneurysm location; D: Hunt-Hess grade; E: Fisher grade; F: intraventricular hemorrhage; G: acute hydrocephalus; H: intracerebral hemorrhage; I: intracranial infection; J: multiple bleeding; K: treatment modality; L: clinical vasospasm; M: hypertension; N: rebleeding; O: external ventricular drainage; P: diabetes mellitus; Q: mechanical ventilation

**Fig. 1:**
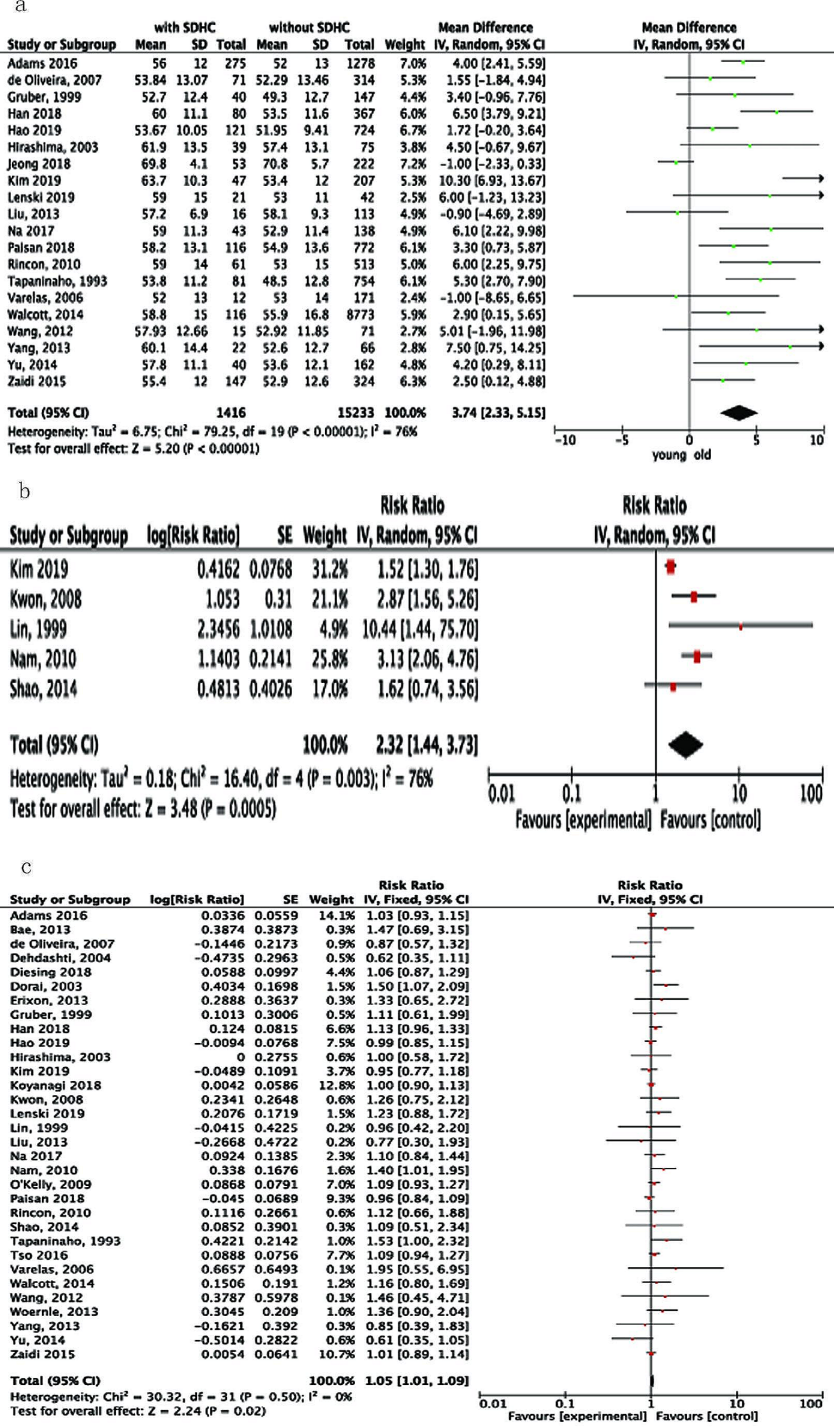
Forest plots for the relation between SDHC and (**A**) age; (**B**) age>50 yrs versus ≤ 50 yrs; and (**C**) gender. Diamonds represent pooled estimates, and the width of the diamonds represents 95%CIs.

Thirty studies reported the average age of patients in both the SDHC and non-SDHC groups, but only 20 of them provided the standard deviation [2, 14, 15, 19, 23, 26–28, 30, 32–36, 38–40, 42, 43, 45], with multivariate analysis performed in twelve studies [13, 15, 16, 23, 35, 36, 38–40, 42, 43, 45]. Meta-analysis of 20 studies revealed that patients that developed SDHC had a higher mean age in comparison to those without SDHC (*p* < 0.00001). Further analysis was carried out to compare the incidence of SDHC in patients who were older than 50 years with that of those who were not older than 50 years. Four studies [16, 18, 20, 25, 40] were included and the results showed patients older than 50 years had a more than 2-fold risk of developing SDHC than those who were younger (RR, 2.32; 95%CI: 1.44–3.73*P* = 0.0005; Fig. 1A and B; Table 2).

**Table 2:** Summary of meta-analysis results of risk factors for SDHC

Items	Test for Heterogeneity		Analysis Model	Test for Overall Effect		RR or WMD 95%CI
	I^2^	***P***		Z	***P***	
Age	76%	< 0.00001	Random	5.20	< 0.00001	3.74 (2.33,5.15)
Gender	0%	0.50	Fixed	2.24	0.02	1.05 (1.01,1.09)
Hypertension	48%	0.03	Fixed	5.61	< 0.00001	1.35 (1.22,1.50)
Diabetes mellitus	75%	0.0006	Random	0.09	0.93	0.98 (0.58,1.65)
Required ventilation	67%	0.05	Random	7.63	< 0.00001	2.37 (1.90,2.96)
Rebleeding	0%	0.85	Fixed	4.72	< 0.00001	2.43 (1.68,3.50)
Location of aneurysm	37%	0.13	Fixed	3.35	0.0008	1.66 (1.24,2.24)
Hunt-Hess grade	73%	< 0.00001	Random	10.41	< 0.00001	2.18 (1.88,2.52)
Fisher grade	82%	< 0.00001	Random	7.49	< 0.00001	1.77 (1.52,2.05)
IVH	88%	< 0.00001	Random	9.53	< 0.00001	2.16 (1.84,2.53)
ICH	46%	0.04	Fixed	4.52	< 0.00001	1.27 (1.14,1.41)
Clinical vasospasm	74%	< 0.00001	Random	3.87	= 0.0001	1.53 (1.24,1.90)
Intracranial infection	35%	0.14	Fixed	18.09	< 0.00001	4.63 (3.92,5.47)
Acute hydrocephalus	93%	< 0.00001	Random	9.40	< 0.00001	3.15 (2.48,4.01)
EVD	86%	< 0.00001	Random	11.26	< 0.00001	3.52 (2.83,4.39)
Treatment modality	45%	0.04	Random	0.42	0.67	0.96 (0.82,1.14)

Gender was reported in 32 of the 37 included studies [2, 10–16, 18–21, 23, 25–30, 32–38, 40–45], and threeteen [13, 15, 16, 35–38, 40–45] studies used multiple logistic analysis to investigate the independent factors. The pooled data showed that the risk of SDHC was higher in females (RR, 1.05; 95%CI: 1.01–1.09; *P* = 0.02; Fig. 1C) with no evidence of significant heterogeneity.

**Fig. 2:**
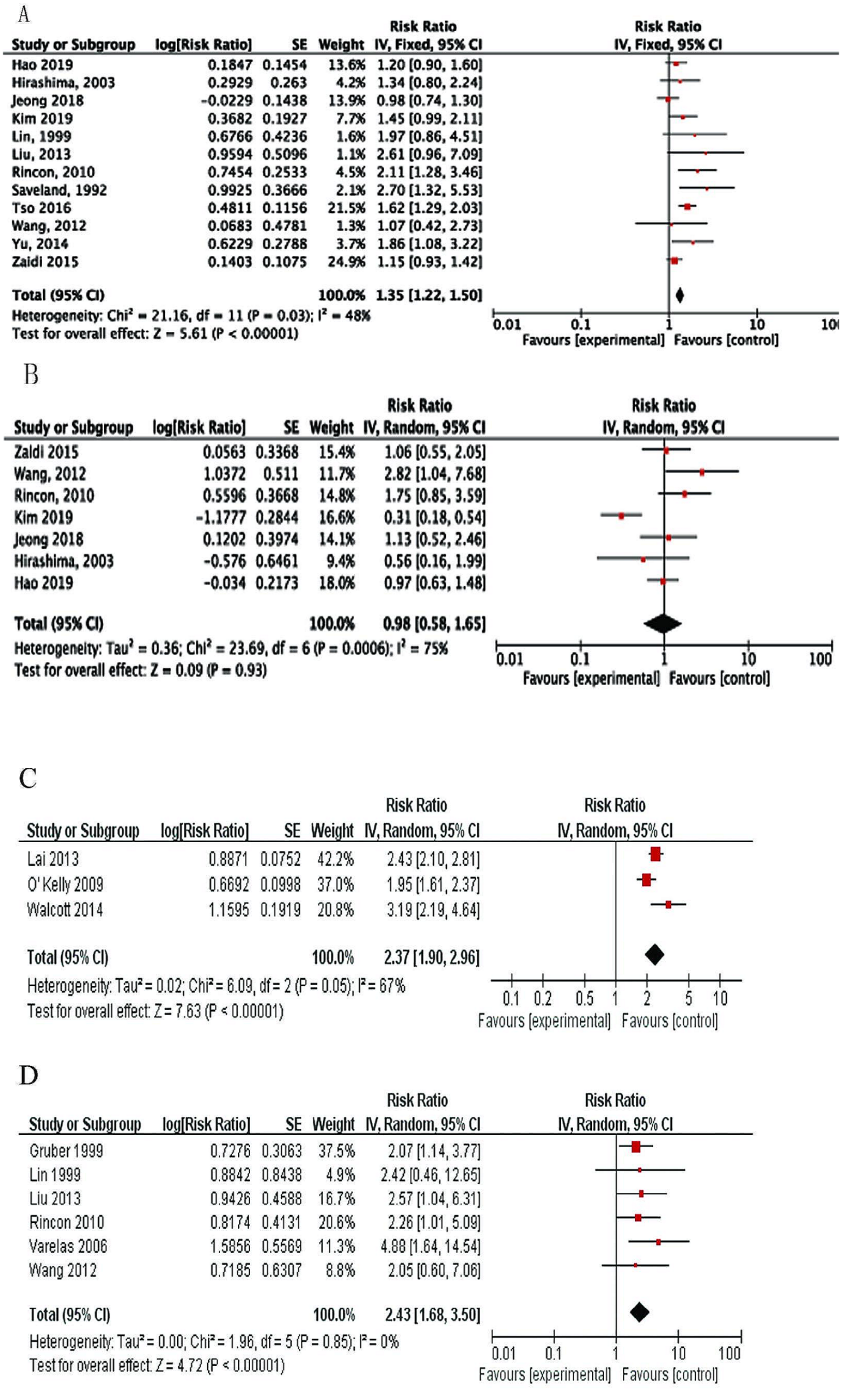
Forest plots for the relation between SDHC and (**A**) preexisting hypertension; (**B**) diabetes mellitus; (**C**) mechanical ventilation; and (**D**) rebleeding. Diamonds represent pooled estimates, and the width of the diamond represents the 95%CI.

Pre-existing hypertension was investigated in twelve studies [15, 18, 19, 23, 24, 28, 32, 38–40, 44, 45] and a meta-analysis found a significant association between hypertension and SDHC development (RR, 1.35; 95%CI: 1.22–1.50; *P* < 0.00001). (Fig. 2A).

Diabetes mellitus (DM) was reported in seven studies [15, 23, 28, 38–40, 45]. The meta-analysis showed no significant association between DM and SDHC (RR, 0.98; 95%CI: 0.58–1.65; *P* = 0.93; Fig. 2B).

Patients who required mechanical ventilation tended to have more SDHC. The pooled data of three included studies [17, 21, 33] revealed a significant association between need for mechanical ventilation and SDHC (RR 2.37; 95%CI: 1.90–2.96; *P* < 0.00001; Fig. 2C).

Six studies [14, 18, 19, 23, 27, 28] investigated the relationship between rebleeding of aSAH and SDHC. One logistic regression analysis showed that rebleeding was an independent risk factor [27]. The rate of SDHC was higher in the rebleeding group (28 of 101) than that of the non-rebleeding group (136 of 1205). The meta-analysis showed over 2-fold increased risk for SDHC (RR 2.43, 95%CI: 1.68–3.50; *P* < 0.00001; Fig. 2D).

**Fig. 3:**
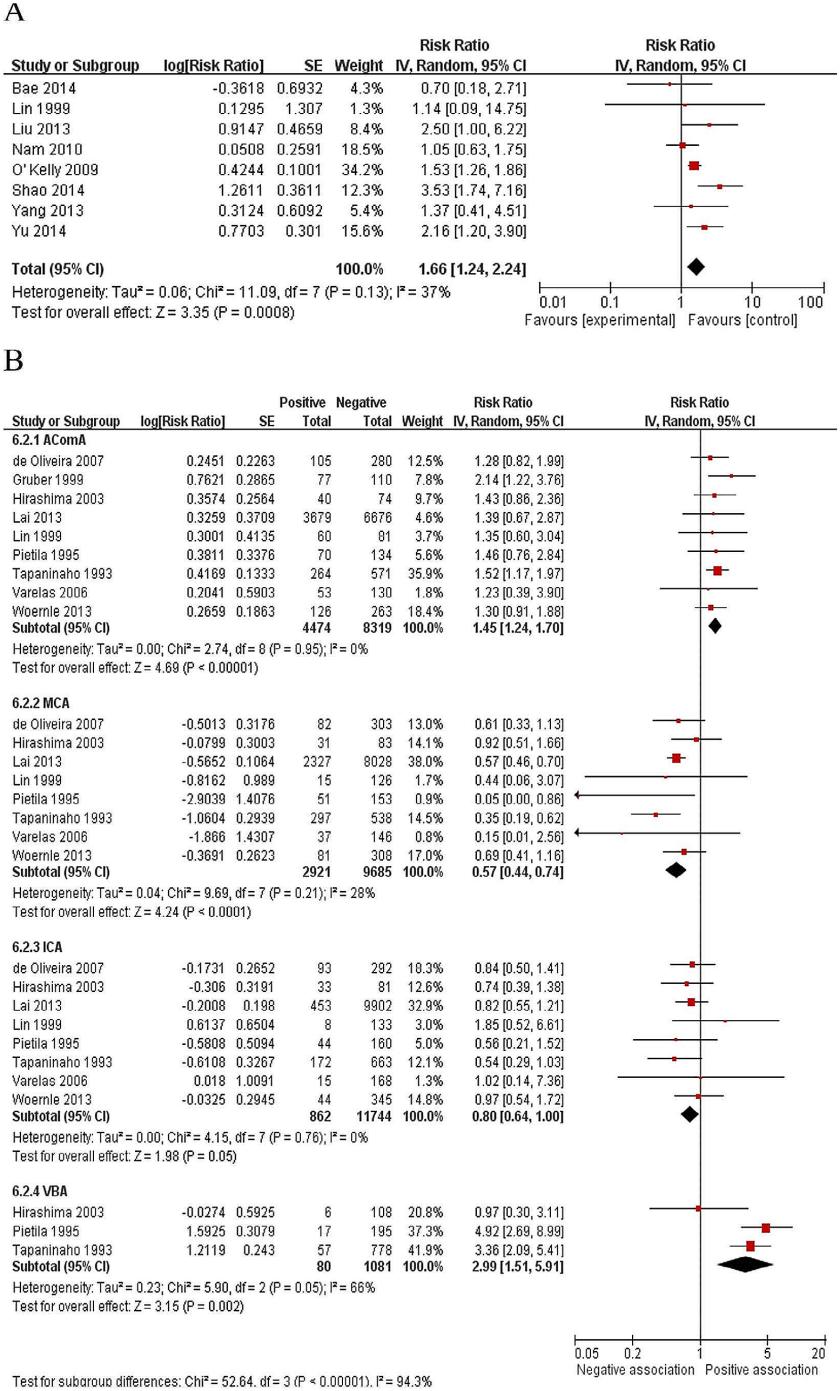
Forest plots for (**A**) the relation between SDHC and ruptured aneurysm location and (**B**) the subgroup of ruptured aneurysm location. Diamonds represent pooled estimates, and the width of the diamond represents the 95%CI.

A total of 30 studies assessed the relationship between ruptured aneurysm location and SDHC, and eight of them [10, 18–21, 25, 30, 32] stratified analysis by the location of the aneurysm (anterior or posterior circulation). Anterior circulation was related to the internal carotid artery (ICA), middle cerebral artery (MCA), anterior communicating artery (AComA) and posterior communicating artery (PComA). Posterior circulation was related to the basilar artery (BasiA), vertebral artery (VBA), and posterior cerebral arteries (PCAs). The pooled data from these eight studies showed a significant association with SDHC (RR 1.66, 95%CI: 1.24–2.24). Subgroup analysis was performed for different arteries, including AComA, MCA, ICA and VBA. The pooled data showed both VBA and AComA was associated with increased risk for developing SDHC (RR, 2.99; 95%CI: 1.51–5.91; *P* = 0.002 and RR, 1.45; 95%CI: 1.24–1.70; *P* < 0.00001, respectively). Neither the MCA nor ICA was associated with development of SDHC (Fig. 3A and B).

**Fig. 4:**
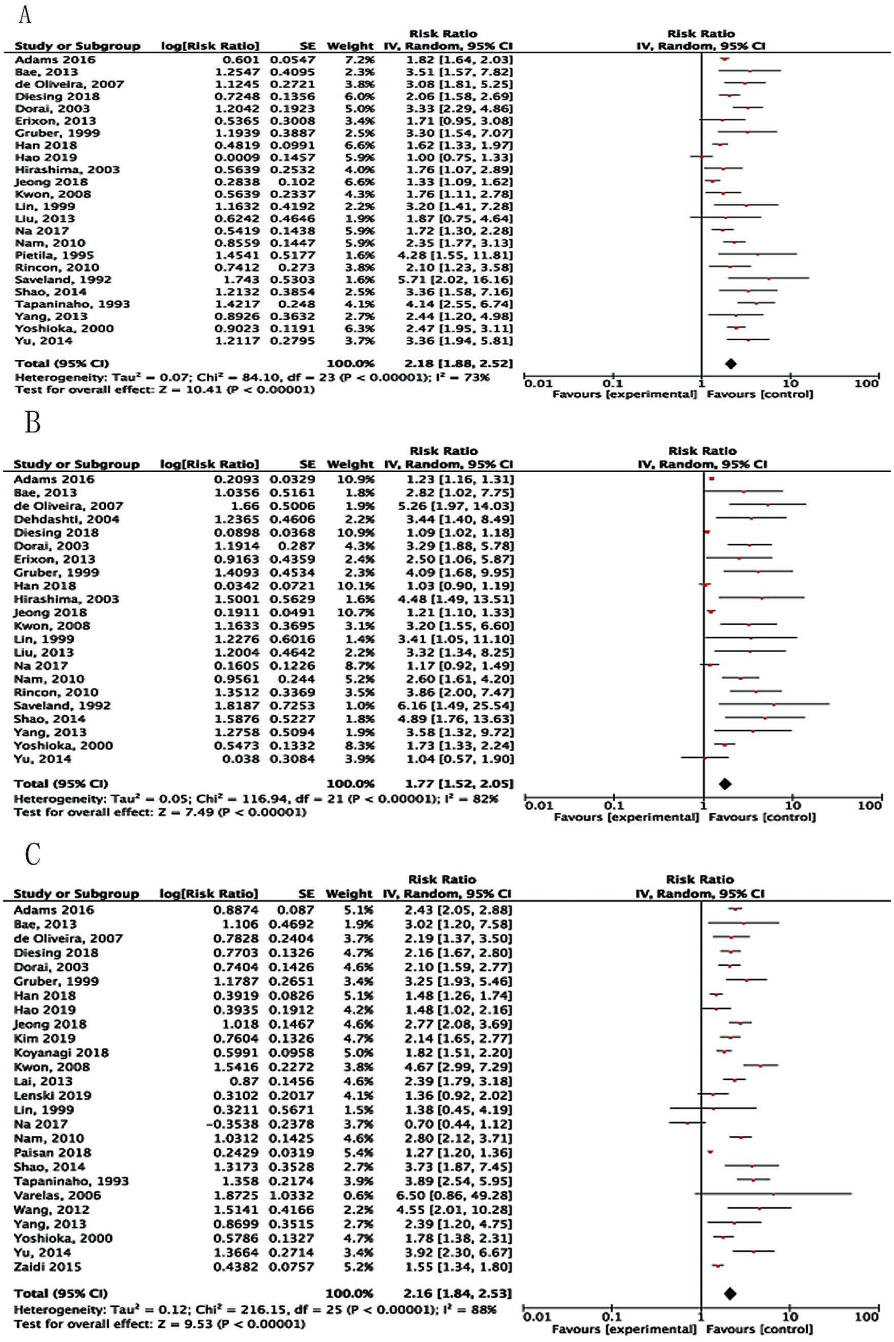
Forest plots for the relation between SDHC and (**A**) Hunt-Hess grade; (**B**) Fisher grade; and (**C**) intraventricular hemorrhage. Diamonds represent pooled estimates, and the width of the diamond represents the 95%CI.

The Hunt and Hess grade was measured after admission, with higher grades indicating poorer neurological status. 24 studies [2, 10, 12–16, 18–20, 22–26, 30–32, 35–39, 42] measured the Hunt and Hess grade and the results of the meta-analysis confirmed that higher Hunt and Hess grades (III-V) were a risk factor for SDHC (RR, 2.18; 95%CI: 1.88–2.52; *P* < 0.00001; Fig. 4A).

The Fisher grade was used to evaluate the severity of SAH according to admission CT appearance [46]. 22 studies [2, 10–16, 18–20, 23–25, 30–32, 35–37, 39, 42] investigating the relation between the Fisher grade and SDHC were included in the meta-analysis. In total, 2,742 patients were pooled into a good condition (grade I–II) group, and the other 5372 patients who were in poor condition (grade III–IV), were put in a poor condition group. The pooled data of these 22 studies showed Fisher grades III–IV had a nearly 2-fold increased risk when compared with grades I–II (RR, 1.77; 95%CI: 1.52–2.05; *P* < 0.00001; Fig. 4B).

The presence of intraventricular hemorrhage (IVH) was investigated in 26 studies [2, 10, 12, 14, 16–18, 20, 25–28, 30–32, 34–43, 45] and twelve multivariate analyses [2, 17, 35–43, 45] found it was an independent risk factor. The pooled result showed 1,081 of 3,939 patients with IVH developed SDHC. The meta-analysis found IVH increased the risk of developing SDHC (RR, 2.16; 95%CI: 1.84–2.53; *P* < 0.00001) with high heterogeneity (I^2^ = 88%; Fig. 4C).

**Fig. 5:**
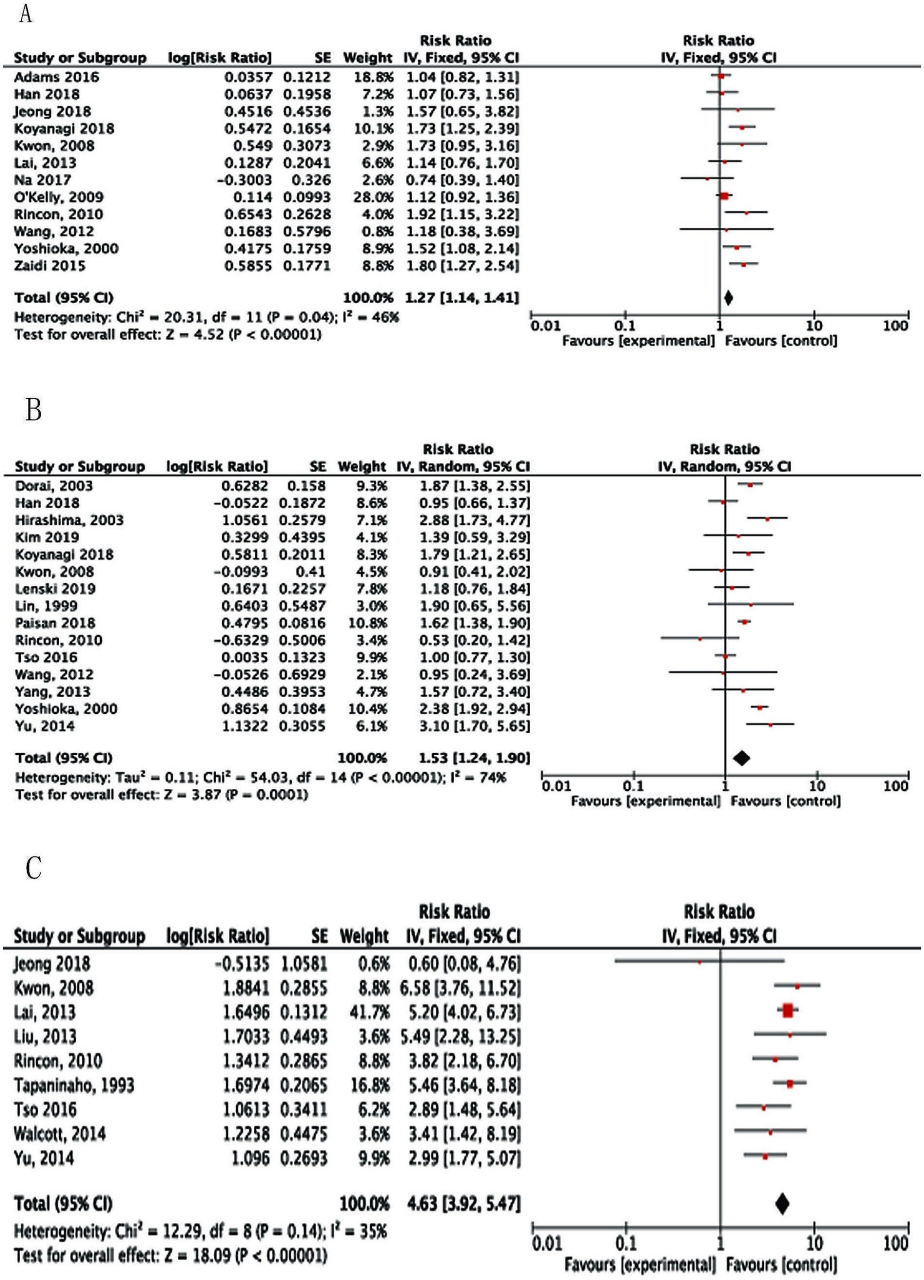
Forest plots for the relation between SDHC and (**A**) ICH; (**B**) clinical vasospasms; and (**C**) intracranial infection. Diamonds represent pooled estimates, and the width of the diamond represents the 95%CI

Twelve studies [16, 17, 21, 23, 28, 31, 35, 36, 39, 41, 42, 45] reported intracerebral hematoma (ICH) according to initial CT examination. Among six studies which could be calculated, [16, 17, 21, 23, 28, 31] 17% (169 of 1,005) of patients with ICH and 10% (1,444 of 14,844) of patients without ICH underwent shunt surgery. The pooled data indicated ICH was a risk factor for SDHC (RR, 1.27; 95%CI: 1.14–1.41; *P* < 0.00001; Fig. 5A).

Patients who demonstrated delayed ischemic neurological deficits and angiographic vasospasms were identified as having clinical vasospasms. Fifteen studies [12, 15, 16, 18, 23, 28, 30–32, 34, 35, 40, 41, 43, 44] investigated the incidence of clinical vasospasms after aSAH. The pooled data of these nine studies indicated that vasospasms appeared to be a significant risk factor for SDHC (RR, 1.53; 95%CI: 1.24–1.90; *P* = 0.0001; Fig. 5B).

Nine studies [16, 17, 19, 23, 26, 32, 33, 39, 44] investigated the incidence of intracranial infection, with five of them [16, 17, 23, 39, 44] using multivariate analyses. The meta-analysis results showed that intracranial infection increased the risk of SDHC by almost five times (RR, 4.63; 95%CI: 3.92–5.47; *P* < 0.00001; Fig. 5C).

**Fig. 6:**
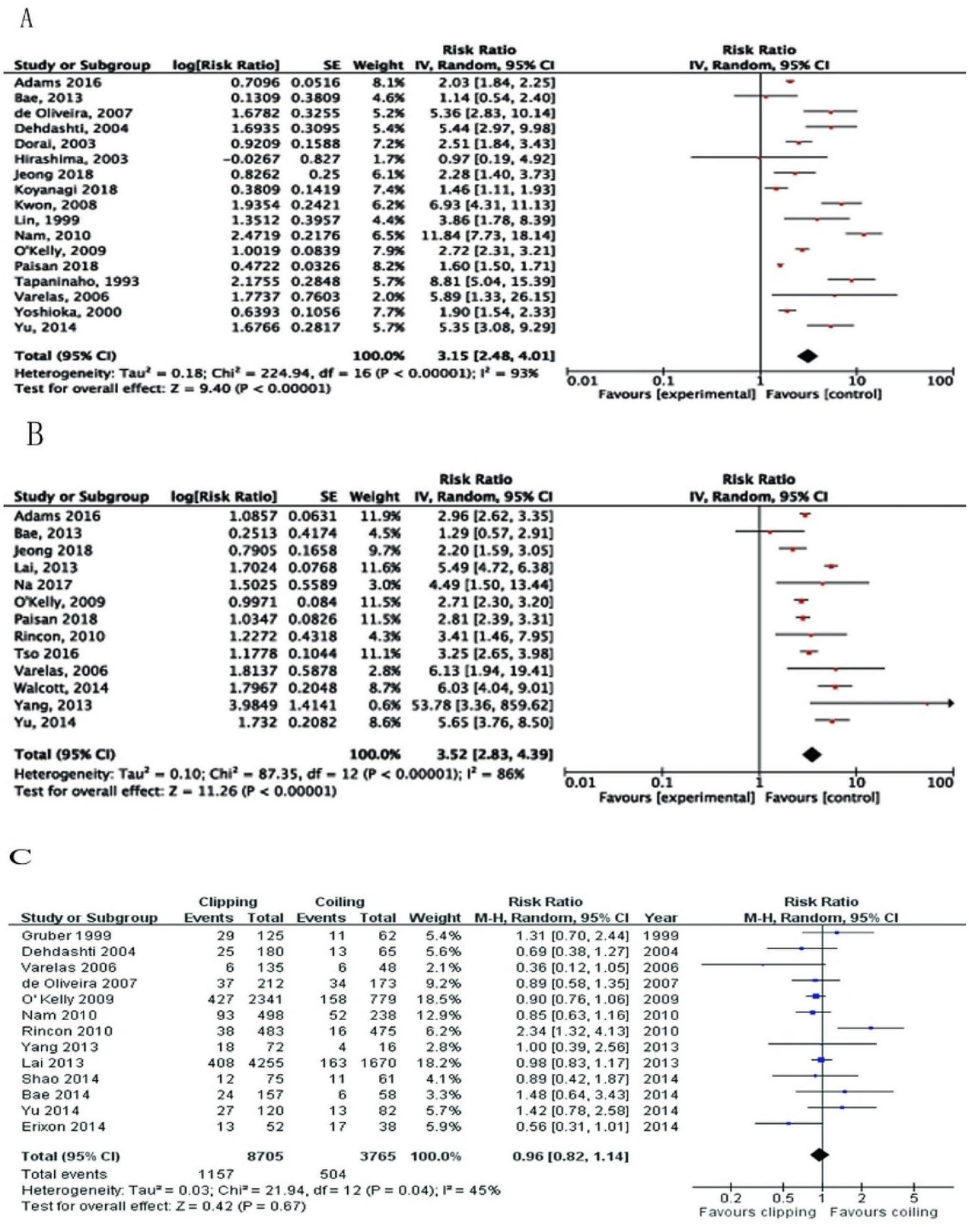
Forest plots for the relation between SDHC and (**A**) Acute hydrocephalus; (**B**) EVD; and (**C**) Treatment modality. Diamonds represent pooled estimates, and the width of the diamond represents the 95%CI

Acute hydrocephalus was reported in 17 studies [2, 10–12, 15, 16, 18, 20, 21, 26, 27, 31, 32, 36, 39, 41, 43] and the pooled data revealed that acute hydrocephalus was related to the incidence of SDHC (RR, 3.15; 95%CI: 2.48–4.01; *P* < 0.00001) with high heterogeneity (I^2^ = 89%; Fig. 6A).

External ventricular drainage (EVD) was placed to drain cerebrospinal fluid for treating early hydrocephalus. 13 studies [10, 17, 21, 23, 27, 30, 32, 33, 36, 39, 42–44] reported the relation between EVD and SDHC, with multivariate analyses used in seven studies [17, 27, 36, 39, 42–44]. Meta-analysis of these 13 studies showed patients who had EVD placement were more likely to develop SDHC (RR, 3.52; 95%CI: 2.83–4.39; *P* < 0.00001, I^2^ = 86%; Fig. 6B).

Effect of treatment modality (i.e., surgical clipping and endovascular coiling) on SDHC was examined in 21 publications [2, 10, 11, 13, 14, 17, 20, 21, 23, 25, 27, 30, 32, 34, 36–40, 43, 45]. Due to the data in the latest studies are inadequate and can’t be calculated, we analyzed data in 13 publications [2, 10, 11, 13, 14, 17, 20, 21, 23, 25, 27, 30, 32]. There were 1,157 of 8,705 patients in the clipping group and 504 of 3,765 patients in the coiling group who developed SDHC. Analysis of the combined data found no significant difference in SDHC incidence between patients treated with clipping versus coiling (RR, 0.96; 95%CI: 0.82–1.14; *P* = 0.67; Fig. 6C).

### Subgroup and sensitivity analyses

In consideration of the strong evidence of heterogeneity for age (I^2^ = 76%), treatment modality (I^2^ = 45%), Hunt-Hess grade (I^2^ = 73%), Fisher grade (I^2^ = 82%), IVH (I^2^ = 88%), clinical vasospasm (I^2^ = 74%), EVD (I^2^ = 86%) and acute hydrocephalus (I^2^ = 93%), subgroup analyses stratified by study design (retrospective or prospective) and geographic area (Europe, North America or Asia) were conducted. Details are shown in Table 3.

**Table 3:** Subgroup analysis of the included studies

Subgroup	NO. of studies	Weight	Test for Heterogeneity		Summary RR(95%CI)	Test for Overall Effect	
			I^2^	*P*		Z	*P*
**Age**							
Retrospective study	16	79%	80%	< 0.00001	3.89(2.18,5.60)	4.46	< 0.00001
Prospective study	4	21%	11%	0.34	3.09(1.36,4.82)	3.50	0.0005
Total	20	100%	76%	< 0.00001	3.74 (2.33,5.15)	5.20	< 0.00001
Europe	5	25.3%	0%	0.5	3.98 (2.79,5.17)	6.54	< 0.00001
North America	5	25.7%	0%	0.45	3.16 (1.81,4.51)	4.59	< 0.00001
Asia	10	49%	86%	< 0.00001	4.15 (1.48,6.83)	3.04	0.002
Total	20	100%	76%	< 0.00001	3.74 (2.33,5.15)	5.20	< 0.00001
**Fisher grade**							
Retrospective study	16	86.7%	80%	< 0.00001	1.50(1.30,1.72)	5.72	< 0.00001
Prospective study	6	13.3%	0%	0.87	3.82(2.65,5.46)	7.27	< 0.00001
Total	22	100%	82%	< 0.00001	1.77 (1.52,2.05)	7.49	< 0.00001
Europe	7	31.6%	83%	< 0.00001	1.56 (1.24,1.96)	3.77	0.0002
North America	2	7.8	0%	0.72	3.52 (2.29,5.40)	5.76	< 0.00001
Asia	13	60.6%	78%	< 0.00001	1.77 (1.41,2.21)	4.95	< 0.00001
Total	22	100%	82%	< 0.00001	1.77 (1.52,2.05)	7.49	< 0.00001
**Hunt-Hess grade**							
Retrospective study	19	85%	76%	< 0.00001	2.11 (1.80,2.47)	9.23	< 0.00001
Prospective study	5	15%	27%	0.24	2.61 (1.86,3.66)	5.53	< 0.00001
Total	24	100%	73%	< 0.00001	2.18 (1.88,2.52)	10.41	< 0.00001
Europe	8	30.3%	68%	0.003	2.60 (1.98,3.43)	6.82	< 0.00001
North America	2	8.8%	48%	0.17	2.75 (1.76,4.31)	4.45	< 0.00001
Asia	14	61%	74%	< 0.00001	1.95 (1.60,2.38)	6.65	< 0.00001
Total	24	100%	73%	< 0.00001	2.18 (1.88,2.52)	10.41	< 0.00001
**Treatment modality**							
Retrospective study	9	66.5%	64%	0.004	0.85 (0.68,1.06)	1.43	0.15
Prospective study	5	33.5%	73%	0.005	1.01 (0.62,1.63)	0.04	0.97
Total	14	100%	66%	0.0003	0.90 (0.74,1.10)	1.04	0.30
Europe	5	39.9%	25%	0.25	0.90 (0.73,1.11)	0.99	0.32
North America	4	31.2%	90%	< 0.00001	0.79 (0.41,1.54)	0.69	0.49
Asia	5	28.9%	0%	0.52	0.98 (0.77,1.24)	0.19	0.85
Total	14	100%	66%	0.0003	0.90 (0.74,1.10)	1.04	0.30
**IVH**							
Retrospective study	23	87.7%	89%	< 0.00001	2.18 (1.82,2.60)	8.53	< 0.00001
Prospective study	3	12.3%	77%	0.01	2.11 (1.35,3.30)	3.27	0.001
Total	26	100%	88%	< 0.00001	2.16 (1.84,2.53)	9.53	< 0.00001
Europe	7	29.5%	59%	0.02	2.37 (1.95,2.87)	7.62	< 0.00001
North America	4	15.7%	84%	0.0004	1.59 (1.24,2.03)	3.68	0.0002
Asia	15	54.8%	82%	< 0.00001	2.21 (1.78,2.75)	7.15	< 0.00001
Total	26	100%	88%	< 0.00001	2.16 (1.84,2.53)	9.53	< 0.00001
**Clinical vasospasm**							
Retrospective study	14	96.6%	74%	< 0.00001	1.59 (1.29,1.97)	4.27	< 0.00001
Prospective study	1	3.4%	-	-	0.53 (0.20,1.42)	1.26	0.21
Total	15	100%	74%	< 0.00001	1.53 (1.24,1.90)	3.87	= 0.0001
Europe	1	7.8%	-	-	1.18 (0.76,1.84)	0.74	0.46
North America	4	33.4%	81%	0.001	1.31 (0.92,1.87)	1.51	0.13
Asia	10	58.8%	68%	0.0009	1.73 (1.27,2.35)	3.49	0.0005
Total	15	100%	74%	< 0.00001	1.53 (1.24,1.90)	3.87	= 0.0001
**EVD**							
Retrospective study	11	84.5%	89%	< 0.00001	3.58 (2.77,4.62)	9.76	< 0.00001
Prospective study	2	15.5%	0%	0.91	3.26 (2.67,3.97)	11.63	< 0.00001
Total	13	100%	86%	< 0.00001	3.52 (2.83,4.39)	11.26	< 0.00001
Europe	2	23.5%	97%	< 0.00001	4.02 (2.20,7.37)	4.52	< 0.00001
North America	6	50.0%	68%	0.007	3.34 (2.70,4.13)	11.15	< 0.00001
Asia	5	26.5%	81%	0.0003	3.41 (1.69,6.87)	3.43	0.0006
Total	13	100%	86%	< 0.00001	3.52 (2.83,4.39)	11.26	< 0.00001
**Acute hydrocephalus**							
Retrospective study	15	89.4%	93%	< 0.00001	2.95 (2.31,3.78)	8.62	< 0.00001
Prospective study	2	10.6%	0%	0.97	5.40 (3.48,8.38)	7.52	< 0.00001
Total	17	100%	93%	< 0.00001	3.15 (2.48,4.01)	9.40	< 0.00001
Europe	4	24.3%	93%	< 0.00001	4.65 (2.04,10.61)	3.65	0.0003
North America	4	25.3%	93%	< 0.00001	2.33 (1.56,3.49)	4.12	< 0.00001
Asia	9	50.4%	92%	< 0.00001	3.02 (1.76,5.18)	4.03	< 0.00001
Total	17	100%	93%	< 0.00001	3.15 (2.48,4.01)	9.40	< 0.00001

Totals in the table represent pooled data from all available studies

- Not applicable

For age, when studies were stratified by study design, retrospective studies still showed significant heterogeneity (I^2^ = 80%), while prospective studies showed insignificant heterogeneity (I^2^ = 11%). When studies were divided by geographic area, the I^2^ of the North American group and European group both decreased to 0%, while high heterogeneity still existed in the Asian group (I^2^ = 86%).

For treatment modality, when studies were stratified by study design, both analyses of retrospective and prospective studies showed significant heterogeneity (I^2^ = 64% and 73%, respectively). When studies were divided by geographic area, the I^2^ of the Asian group and European group decreased to 0% and 25%, respectively, while high heterogeneity still existed in the North American group (I^2^ = 90%).

For Fisher grade, heterogeneity was not detected in the prospective group and North American group. For Hunt-Hess grade, the I^2^ of the prospective group and North American group decreased to 27% and 48%, respectively.

Neither study design nor geographic area was the heterogeneity source for IVH and clinical vasospasms. All subgroup analyses showed significant heterogeneity.

Analysis of studies stratified by study design found a relatively high heterogeneity in retrospective studies for EVD (I^2^ = 89%) compared with the total studies (I^2^ = 87%). For prospective studies, the I^2^ decreased to 0%. While North American subgroup analysis found a slightly decreased heterogeneity (I^2^ = 68%) with a combined RR of 3.34 (95%CI: 2.70–4.13).

For acute hydrocephalus, when the studies were stratified by study design, heterogeneity was eliminated in the prospective group (I^2^ = 0%). When the studies were divided according to geographic area, the heterogeneity still remained at a relatively high level for all the three subgroup (I^2^ = 93%, 93%, and 92%, respectively).

Sensitivity analysis was performed by leaving the included study out one by one if the heterogeneity was unacceptable. For age, Hunt-Hess grade, Fisher grade, IVH, clinical vasospasms, EVD and acute hydrocephalus, the pooled results were not significantly attenuated after excluding every study.

### Publication bias

Funnel plots of standard error (SE) versus RR were constructed for factors with more than 10 studies. For gender, visual inspection of the funnel plots did not show any remarkable asymmetry. However, for treatment modality, Fisher grade, Hunt-Hess grade, IVH and acute hydrocephalus, a asymmetry was found at the bottom of the funnel, which indicated that possible publication bias.

## Discussion

aSAH is one of the most common cerebrovascular diseases. SDHC is a well-recognized complication that burdens patients suffering from aSAH physically and economically. To our knowledge, this is the first systematic review and meta-analysis with strict inclusion criteria to investigate risk factors associated with SDHC after aSAH.

According to our research, the incidence of SDHC was related to multiple factors. Elderly patients are more likely to have SDHC, perhaps because they usually have larger ventricles that tend to have a diffuse collection of subarachnoid blood. In addition, elderly patients often have more chronic diseases that could promote the development of SDHC. For instance, chronic pulmonary disease has been shown to have a connection with the incidence of SDHC. [26]

Female sex is a significant risk factor for SDHC. Most studies have identified a higher incidence of SAH in the female population. Algra et al. inferred this phenomenon was associated with female hormones, but the pathophysiology of how hormones lead to the difference between the sexes still remained unclear [47]. Further studies on female hormone regulation may explain the relationship between SDHC and female sex.

Generally, CT scans are used to evaluate SAH severity clinically. Both high Fisher grades and presence with IVH were found to be associated with the development of SDHC. This can be easily understood considering that blood in the ventricle or subarachnoid space obstructs CSF circulation, which could accelerate SDHC development.

Patients who require mechanical ventilation usually are in poor condition with several comorbidities, which may accelerate the development of SDHC. Our meta-analysis supported this phenomenon. Poor Hunt and Hess grades may be secondary to high Fisher grades. Several authors have reported that poor Hunt and Hess grades predicted a devastating outcome after aSAH, and our research supported that conclusion. Poor Hunt and Hess grades are considered as significant risk factors.

In 1986, Black reported an association between hydrocephalus and vasospasms after SAH and speculated that vasospasms could, by presently unknown mechanisms, potentiate hydrocephalus [48]. In our analysis, we concluded that vasospasms are associated with SDHC.

Rates of SDHC were compared between aneurysms within the anterior and posterior circulations. The results indicated that anterior circulation aneurysms are associated with a lower rate of SDHC, which is consistent with previous studies. Controversies exist whether aneurysms of MCA contribute to SDHC. Our subgroup analysis of specific arteries revealed that either aneurysms of the MCA or ICA were not related to a higher incidence of SDHC.

Treatment modality has been investigated by many authors. In a previous meta-analysis conducted by de Oliveira including 1,718 patients, the risk of shunt-dependent hydrocephalus was higher after coiling than after clipping of ruptured intracranial aneurysms [2]. However, our study did not find increased risk for SDHC in patients treated with coiling compared those treated with clipping. We also noted that none of the included studies was randomized and there was moderate heterogeneity; therefore, bias may have existed.

Recently, another meta-analysis study conducted by Zhiyi Xie et al. [49]. indicated that increased age, female gender, high Hunt-Hess grades, low GCS scores (GCS ≤ 8), high Fisher grades (Fisher grade ≥ 3), acute hydrocephalus, EVD insertion, IVH, post circulation aneurysm, AcomA aneurysm, clinical vasospasm, meningitis, and rebleeding were predictors of SDHC following aSAH. However, it only included limited studies, and did not make the subgroup analysis to solve the problems of high heterogeneity. On the contrary, our meta-analysis has advantages in including more comprehensive and updated researches with a larger sample size. Besides, concerning high heterogeneity, our study conducted subgroup analyses stratified by study design (retrospective or prospective) and geographic area (Europe, North America or Asia), which can make our results more precise and persuasive.

There are some limitations in our meta-analysis. Firstly, it seems impossible for neurosurgeons to conduct randomized controlled trials on patients who suffer from aSAH because of ethical reasons. The studies we selected were all cohort studies, whose design lacked a blinded or random allocation of treatment to different groups. Secondly, publication bias cannot be ignored because the published studies our analysis was based on tended to report positive results rather than negative results. Thirdly, many studies did not present adjusted analyses and we pooled the data from both multivariate and univariate models without restriction. As a result, we could not exclude all confounding factors that had effects on the factors we measured. Fourthly, most studies were performed retrospectively and not all the confounders were accounted for in a retrospective design, as a result, we could not draw the conclusion that those risk factors were causal factors.

## Conclusion

In conclusion, this systematic review and meta-analysis found multiple risk factors for SDHC. By understanding the factors related to the development of SDHC, it is expected that early diagnoses will be made and appropriate management of aSAH will result in better prognosis.

## Data Availability

No datasets were generated or analysed during the current study.
